# Serum 25-Hydroxyvitamin D Status Across Vertebrate Species: A Comparative Review

**DOI:** 10.3390/ijms27188333

**Published:** 2026-09-19

**Authors:** Alicja Ciesielska, Dominika Rozmus, Anna Cieślińska

**Affiliations:** Department of Biochemistry, University of Warmia and Mazury, 10-719 Olsztyn, Poland; alicja.ciesielska@doktorant.uwm.edu.pl (A.C.); dominika.rozmus@uwm.edu.pl (D.R.)

**Keywords:** vitamin D status, vertebrates, companion animals, 25-hydroxyvitamin D, livestock, wildlife

## Abstract

Vitamin D plays a fundamental role in calcium and phosphate homeostasis, skeletal development, immune regulation, and numerous metabolic processes in both humans and animals. Serum 25-hydroxyvitamin D [25(OH)D] is the accepted biomarker of vitamin D status, but its physiological concentrations vary considerably among animal species due to differences in metabolism, diet, ultraviolet B (UVB) exposure, and environmental conditions. This review summarizes current knowledge on vitamin D status across vertebrate species and identifies the major determinants responsible for interspecies variation. A structured literature search was conducted using Google Scholar, PubMed, Scopus, and Web of Science. Studies reporting serum or plasma 25(OH)D concentrations in companion animals, livestock, wildlife, reptiles and fish were evaluated, together with publications addressing vitamin D metabolism, analytical methods, and supplementation. Available evidence demonstrates marked differences in serum 25(OH)D concentrations among vertebrate species. Vitamin D status is primarily influenced by UVB exposure, dietary intake, husbandry conditions, season, and species-specific metabolic adaptations. In companion animals such as dogs and cats, dietary intake is the predominant source of vitamin D, whereas in livestock both UVB exposure and supplementation substantially influence circulating 25(OH)D concentrations. Wild animals often show greater seasonal variation in reported 25(OH)D concentrations, although comparisons with domestic counterparts are complicated by differences in diet, supplementation, environmental exposure, and analytical methods. Across multiple species, supplementation with 25-hydroxyvitamin D_3_ appears more effective than cholecalciferol in increasing circulating 25(OH)D concentrations. Reported vitamin D status differs among vertebrate species and cannot be interpreted using universal reference values, particularly when analytical methods and study conditions differ. Species-specific physiology, environmental factors, and nutritional management should all be considered when evaluating serum 25(OH)D concentrations. Future studies should prioritize standardized analytical methodologies, establish species-specific reference intervals, and expand research on wildlife to improve understanding of vitamin D physiology across vertebrates.

## 1. Introduction

### 1.1. Vitamin D Sources and Biological Functions

Vitamin D is a group of fat-soluble secosteroids that includes two major forms: cholecalciferol (vitamin D_3_) and ergocalciferol (vitamin D_2_) [1]. Vitamin D_3_ is synthesized in the skin following ultraviolet B (UVB)-induced photochemical conversion of 7-dehydrocholesterol, an intermediate in cholesterol biosynthesis, whereas vitamin D_2_ is produced primarily by plants, fungi, and yeasts [2,3]. Both forms undergo sequential hydroxylation in the liver and kidneys to generate the biologically active metabolite 1,25-dihydroxyvitamin D, which exerts its effects through the vitamin D receptor (VDR) [4].

The active form of vitamin D regulates of calcium and phosphate metabolism, bone mineralization, and immunological and metabolic processes [5,6]. Increasing evidence also supports its involvement in brain development, cognitive function, and neuroprotection [7]. Vitamin D deficiency is now observed globally in both humans [8] and animals [9].

Serum 25-hydroxyvitamin D (25OHD, calcidiol), the major circulating metabolite of vitamin D, is widely recognized as the most reliable biomarker of vitamin D status due to its relatively long half-life and its ability to reflect both endogenous synthesis and dietary intake [10]. In animals, adequate vitamin D status is essential for skeletal health, immune competence, and normal metabolic function [9,11]. Vitamin D is also important from a nutritional perspective, because animal-derived foods such as meat, milk, eggs, and fish are major dietary sources of vitamin D for humans [12]. Although reference values for serum 25OHD have been extensively investigated in humans, they remain poorly defined for many animal species. Differences in vitamin D metabolism, husbandry practices, dietary supplementation, geographical location, and exposure to ultraviolet radiation further complicate comparisons across species. Therefore, a comprehensive evaluation of serum 25OHD concentrations in domestic, companion, laboratory, and wild animals is needed to improve our understanding of species-specific vitamin D physiology and its implications for veterinary medicine, animal welfare, and human nutrition.

Vitamin D exists in two major forms: vitamin D_3_ (cholecalciferol) and vitamin D_2_ (ergocalciferol). Vitamin D_3_ is synthesized in the skin following exposure to UVB radiation and is also obtained from foods of animal origin, particularly fatty fish, eggs, and dairy products. In contrast, vitamin D_2_ is derived mainly from plants and fungi [13].

### 1.2. Vitamin D Metabolism

After entering the circulation, vitamin D undergoes hydroxylation in the liver to form 25(OH)D, the major circulating form of vitamin D [2]. A second hydroxylation occurs primarily in the kidneys, producing the biologically active metabolite 1,25-dihydroxyvitamin D_3_ [1,25(OH)_2_D_3_, calcitriol] [14]. The activation and degradation of vitamin D metabolites are regulated by cytochrome P450 enzymes. CYP2R1 and CYP27A1 mediate the conversion of vitamin D to calcidiol [25(OH)D], CYP27B1 catalyzes the synthesis of the biologically active calcitriol [1,25(OH)_2_D], whereas CYP24A1 is responsible for the catabolism of both calcidiol and calcitriol into biologically inactive metabolites [15]. Owing to its relatively long half-life and stable serum concentration, 25(OH)D is considered the most reliable biomarker of vitamin D status [16].

Calcitriol exerts its biological effects by binding to the vitamin D receptor (VDR), which regulates numerous physiological processes, including calcium and phosphate homeostasis, cell proliferation and differentiation, energy metabolism, and innate and adaptive immune responses [17,18,19].

**Figure 1:**
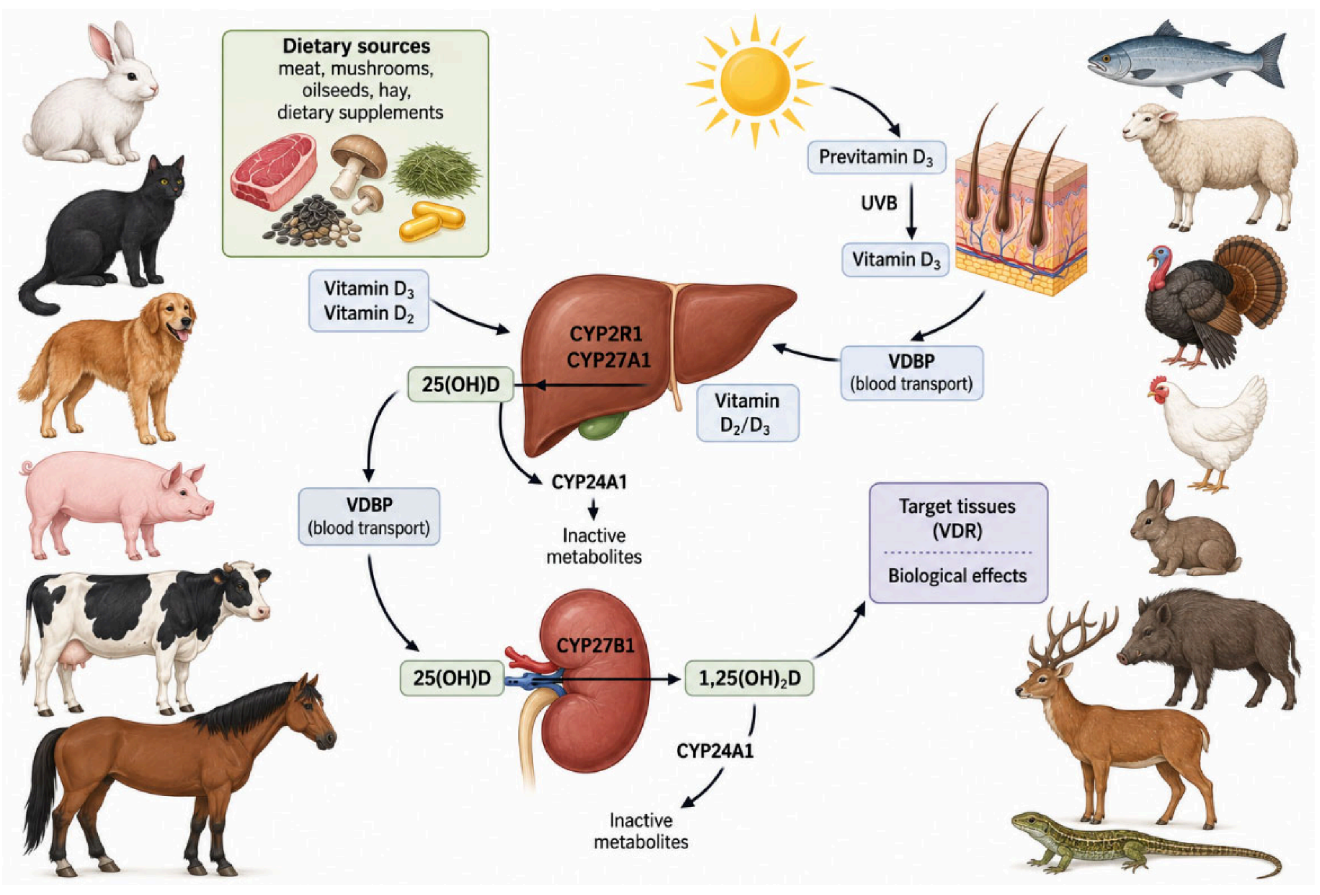
Vitamin D metabolism. UVB, ultraviolet B radiation; VDBP, vitamin D-binding protein; CYP2R1, cytochrome P450 family 2 subfamily R member 1; CYP27A1, cytochrome P450 family 27 subfamily A member 1; CYP24A1, cytochrome P450 family 24 subfamily A member 1; CYP27B1, cytochrome P450 family 27 subfamily B member 1; VDR, vitamin D receptor; 25(OH)D, 25-hydroxyvitamin D; 1,25(OH)_2_D, 1,25-dihydroxyvitamin D; D_2_, vitamin D_2_; D_3_, vitamin D_3_.

Another key component of the vitamin D pathway is vitamin D-binding protein (VDBP), the major transporter of vitamin D metabolites in circulation. Genetic polymorphisms in VDR and VDBP genes may influence vitamin D metabolism, circulating 25(OH)D concentrations, and individual responses to vitamin D [20,21,22,23] (Figure 1).

Importantly, 25(OH)D is not confined solely to the circulation. Skeletal muscle can take up and store 25(OH)D via a VDBP-dependent mechanism, creating an extravascular pool of this metabolite that may contribute to maintaining its concentration in the blood [24,25,26].

VDBP is highly evolutionarily conserved and is found in almost all vertebrates. However, its concentration and the binding properties of vitamin D metabolites vary across species. In most vertebrates, starting with bony fish, VDBP is the primary transport protein for 25(OH)D, although lipoproteins may also perform this function in some fish species. VDBP concentrations in reptiles are significantly lower than in birds and mammals [27]. Vieth et al. [28] demonstrated significant interspecies differences in VDBP affinity for 25(OH)D_3_ and the resulting free 25(OH)D_3_ fraction in rats, horses, cattle, rhesus monkeys, and humans. The lowest fraction of free 25(OH)D_3_ was observed in rats and the highest in humans, with a nearly fivefold difference between the species at comparable total 25(OH)D_3_ concentrations.

Furthermore, studies on mice have shown that similar total concentrations of 25(OH)D_2_ and 25(OH)D_3_ can correspond to different concentrations of free 25(OH)D, a phenomenon linked to differences in bone tissue parameters. Importantly, apart from studies on mice, data regarding free 25(OH)D and its biological significance in other species are very limited [29].

Vitamin D metabolism varies considerably among vertebrate species due to differences in cutaneous synthesis, dietary intake, and adaptation to environmental conditions. Compared with humans, most animals have a limited capacity for skin synthesis of vitamin D because of the presence of hair or feathers and lower concentrations of 7-dehydrocholesterol in the skin [30]. As a consequence, dietary vitamin D is often the primary source of this vitamin. In mammals, vitamin D status depends on both UVB exposure and dietary intake, although their relative importance differs among species. In livestock, including cattle, pigs, and horses, feed supplementation is particularly important in intensive production systems, where limited sunlight exposure may increase the risk of vitamin D deficiency and impaired calcium–phosphorus metabolism [31].

In companion animals, especially dogs and cats, cutaneous vitamin D synthesis is negligible, making dietary intake essential. Besides its well-established role in skeletal health, vitamin D also contributes to immune regulation [11]. In wild mammals, serum vitamin D concentrations are influenced by seasonal sunlight exposure and environmental conditions, with evidence suggesting a contribution of genetic factors to vitamin D status [32]. Historical studies also suggest that UV irradiation of sterol-containing feed can increase its antirachitic activity after ingestion. In fur-bearing animals, ingestion of vitamin D formed on the skin or associated with the fur during grooming has also been proposed, although the contribution of this pathway remains uncertain [33].

In birds, vitamin D plays a crucial role in maintaining calcium and phosphorus homeostasis, skeletal development, and eggshell formation. Because of plumage, skin exposure to UVB radiation is limited, so the diet serves as a significant source of vitamin D. It has been demonstrated that in poultry, supplementation with 25OHD can improve mineral utilization more effectively than supplementation with vitamin D_3_. At the same time, the mechanisms by which birds acquire vitamin D are more complex and also include the possibility of synthesis induced by UVB radiation [34,35,36].

The uropygial gland is the primary cutaneous gland in birds, and its lipid-rich secretion is distributed over the feathers during preening [37]. Early studies suggested that removal of the gland could lead to rickets in chicks despite proper nutrition and exposure to sunlight; this gave rise to the hypothesis that provitamin D_3_ is secreted onto the feathers, where it is converted into vitamin D_3_ by radiation and subsequently ingested during preening [38]. However, subsequent studies did not detect provitamin D in the uropygial glands of chickens, ducks, or geese, whereas its presence was confirmed in the feet; it was also demonstrated that irradiating the feet could elicit an antirachitic effect [39]. Available data thus indicate that the mechanisms of vitamin D acquisition and metabolism in birds are more complex than the initial hypothesis regarding the role of the uropygial gland suggested [40].

In fish, vitamin D metabolism depends largely on dietary intake and differs from that of terrestrial vertebrates because of species-specific regulation of hydroxylating enzymes and a broader tissue distribution of the vitamin D receptor (VDR). These adaptations reflect the distinct mechanisms of calcium homeostasis in the aquatic environment. Experimental studies have also demonstrated beneficial effects of vitamin D supplementation on intestinal function and immune responses in fish [41,42].

The main characteristics of vitamin D acquisition and the factors influencing vitamin D status in companion, livestock, and wild animals are summarized in Table 1.

### 1.3. Assessment of Vitamin D Status

Liquid chromatography–tandem mass spectrometry (LC-MS/MS) is considered the reference method for measuring serum 25(OH)D because of its high accuracy, specificity, and ability to distinguish individual vitamin D metabolites [45]. Although immunoassays are widely used in clinical practice, they are associated with inter-assay variability, analytical bias, and limited comparability, particularly at low 25(OH)D concentrations [46].

Variability among analytical methods remains a major challenge in the assessment of vitamin D status. Differences in assay accuracy and standardization hinder comparisons between laboratories and clinical studies, complicate meta-analyses, and contribute to inconsistent definitions of vitamin D deficiency and sufficiency among scientific societies and public health organizations. This lack of harmonization has been described as “vitamin D guidelines paralysis” [47]. The use of different analytical methods, with varying accuracy, specificity, calibration, and standardization, may affect reported 25(OH)D concentrations. Therefore, numerical differences between animal species should be interpreted cautiously, particularly when analytical methods, biological matrices, study populations, or reporting conventions are not comparable. LC-MS/MS offers high analytical specificity and enables the simultaneous measurement and differentiation of individual vitamin D metabolites, including 25(OH)D_2_ and 25(OH)D_3_. However, LC-MS/MS is not free from analytical limitations, including matrix effects, epimeric and isobaric interferences, and variability related to sample preparation, ionization, and instrumental parameters [48]. Its application in comparative studies has demonstrated its usefulness for assessing vitamin D metabolites across diverse animal species, including non-human primates, birds, reptiles, and mammals [49,50]. Importantly, interspecies validation of LC-MS/MS has been reported for 25(OH)D_3_ and 25(OH)D_2_ in serum from rats, dogs, mice, and monkeys, demonstrating acceptable analytical accuracy and precision across these species [51]. Nevertheless, validation in the specific biological matrix remains important when applying an assay to a new species.

The presence of vitamin D epimers—particularly 3-epi-25(OH)D_3_, which can constitute a significant fraction of the total measured 25(OH)D—represents a crucial analytical issue. Methods that fail to distinguish between 3-epi-25(OH)D_3_ and 25(OH)D_3_ may overestimate results and increase inter-study variability. Chromatographic methods coupled with mass spectrometry allow for the differentiation of specific metabolites and epimers; therefore, the specificity of the method employed must be taken into account when comparing 25(OH)D concentrations across species and studies [52,53,54].

However, epimers may hold physiological significance in addition to their analytical importance. Studies in pigs have demonstrated that supplementation with the 14-epimer of 25(OH)D_3_ influenced circulating 25(OH)D_3_ concentrations and reproductive parameters compared with standard 25(OH)D_3_ and vitamin D_3_, suggesting a potential biological role for these compounds in animals [55]. This highlights the need for a better understanding of vitamin D epimers—especially in wild animals—and the importance of considering method specificity when interpreting results.

### 1.4. Vitamin D Supplementation and Reference Serum 25(OH)D Concentrations in Humans

Vitamin D plays a central role in calcium and phosphate homeostasis; however, the mechanisms regulating its status differ among species. In humans, vitamin D is obtained primarily through UVB-induced skin synthesis and, to a lesser extent, from dietary sources [56,57]. Recommendations regarding optimal serum 25(OH)D concentrations and their assessment have evolved considerably, and current guidelines do not support routine measurement of 25(OH)D or the use of fixed target concentrations in the general population [56,57]. However, the interpretation of studies on vitamin D supplementation should take into account factors such as baseline vitamin D status and the dose–response relationship [58]. Many large randomized trials included individuals with sufficient vitamin D levels, which may have limited the assessment of supplementation benefits in those with a deficiency [59]. More recent analyses also highlight the importance of baseline vitamin D status [60].

Differences among recommendations partly reflect differences in their underlying objectives, particularly regarding skeletal and non-skeletal outcomes [16,56,57,61].

The lack of consensus regarding optimal serum 25(OH)D concentrations and vitamin D supplementation contributes to variability among international recommendations [62].

This review adopts a comparative approach by classifying vertebrate species into companion, livestock, and wild animals according to their typical UVB exposure and reliance on dietary vitamin D. This framework facilitates the evaluation of factors influencing serum 25(OH)D concentrations while accounting for species-specific physiological differences.

## 2. Materials and Methods

A structured literature search was conducted to summarize the current evidence on serum 25(OH)D concentrations across vertebrate species and to identify the major determinants of vitamin D status. A comprehensive literature search was performed using the Google Scholar, PubMed, Scopus, and Web of Science databases. The search included articles published up to March 2026. The following keywords were used: vitamin D, 25-hydroxyvitamin D OR 25(OH)D, calcidiol, vitamin D metabolism, vitamin D status, companion animals, livestock, wildlife, reptiles, fish, birds, mammals, together with species-specific terms (e.g., dog, cat, horse, pig, cattle, sheep, rabbit, wild boar, red deer).

Original research articles constituted the primary source of evidence. Review articles, clinical guidelines, consensus statements, and selected book chapters were additionally consulted to provide background information on vitamin D metabolism, analytical methods, and reference values. Studies reporting serum or plasma 25(OH)D concentrations, vitamin D metabolism, dietary supplementation, analytical aspects of vitamin D determination, or determinants of vitamin D status in vertebrate species were considered eligible. Human studies were included only when discussing vitamin D metabolism, analytical methods, and reference concentrations, providing a framework for interpreting animal data. Editorials, duplicate publications, and studies not relevant to the objectives of this review or lacking sufficient quantitative or methodological information were excluded. The findings were synthesized narratively, with particular emphasis on interspecies differences in serum or plasma 25(OH)D concentrations, determinants of vitamin D status, and methodological factors influencing data interpretation. For each eligible study, the following information was extracted whenever available: animal species, sample size, study design, analytical method used for vitamin D determination, biological material analyzed, reported serum or plasma 25(OH)D concentrations, and the principal factors influencing vitamin D status, including diet, UVB exposure, season, age, and supplementation.

## 3. Model Animals in Science

### 3.1. Mice (Mus musculus)

Mice serve as an important model in research on vitamin D metabolism, and 25(OH)D concentrations can vary depending on strain, age, sex, and vitamin D intake. In Biozzi and C57BL/6 mice, higher 25(OH)D concentrations were observed in older animals, in C57BL/6 mice, and in females [63]. Restricting dietary vitamin D_3_ intake led to a significant reduction in 25(OH)D levels [64]. Furthermore, studies on germ-free mice demonstrated the influence of microbiota on vitamin D metabolism and calcium homeostasis; 25(OH)D and 24,25(OH)_2_D concentrations were determined using LC–MS/MS [65]. These results indicate that mouse models enable the assessment of both the factors influencing vitamin D status and the mechanisms regulating its metabolism.

A crucial component of these mechanisms is the transport of vitamin D_3_ from its site of synthesis into the circulation, a process in which vitamin D-binding protein (VDBP) plays a key role. Studies conducted on mice indicate that vitamin D status is influenced not only by cutaneous synthesis and dietary intake but also by processes occurring after its production. Following UVB exposure, a significant portion of vitamin D_3_ in the skin exists in the form of esters—accounting for approximately 80% of total vitamin D_3_—which undergo hydrolysis prior to transport into the circulation [66]. Vitamin D-binding protein (VDBP) plays a crucial role in this subsequent transport; in mice lacking this protein (DBP−/−), UVB exposure failed to increase serum 25(OH)D or calcium levels despite normal cutaneous vitamin D_3_ synthesis, whereas the response to UVB was restored upon administration of recombinant DBP [67]. Furthermore, it has been demonstrated that cutaneously synthesized vitamin D_3_ exhibits approximately twice the biological activity of an equivalent oral dose, an effect attributed to VDBP-mediated transport and reduced biliary excretion of D_3_ [68]. The results obtained in the mouse model therefore indicate that the concentration of circulating 25(OH)D is the net result not only of vitamin D synthesis and supply but also of its release from the skin, transport by VDBP, and subsequent bioavailability. These factors must be taken into account when interpreting vitamin D status and differences in 25(OH)D concentrations between species.

### 3.2. Rats (Rattus norvegicus)

The rat is a widely used model for studying vitamin D metabolism and calcium–phosphate homeostasis. A model of controlled vitamin D deficiency has been developed in Wistar rats, resulting in reduced concentrations of 25(OH)D_3_ and 1,25(OH)_2_D_3_ while maintaining calcium homeostasis [69]. It has also been shown that lead exposure lowered 25(OH)D concentrations—measured via LC–MS/MS [70]—whereas sunlight exposure and vitamin D_3_ supplementation improved vitamin D status and parameters of calcium–phosphate homeostasis [71]. Furthermore, 25(OH)D concentration correlated with phosphate levels, indicating a close link between vitamin D metabolism and mineral homeostasis [72]. These studies confirm the utility of the rat as a model for vitamin D research, while also highlighting that 25(OH)D levels should be interpreted within the context of calcium–phosphate homeostasis. Although interspecies differences limit the direct extrapolation of results, comparing findings across species can help identify mechanisms shared among vertebrates.

### 3.3. Non-Human Primates

Non-human primates serve as valuable models for vitamin D research due to their close evolutionary relationship with humans, while also allowing for the assessment of interspecies differences. In captive western lowland gorillas (*Gorilla gorilla gorilla*), 25(OH)D concentrations varied according to natural light exposure, averaging 35.5 ± 14.8 nmol/L (14.2 ± 5.9 ng/mL) in animals kept primarily indoors and 66.7 ± 42.0 nmol/L (26.7 ± 16.8 ng/mL) in those with regular access to an outdoor enclosure [73]. A European study involving chimpanzees (*Pan troglodytes*) found 25(OH)D concentrations—measured via LC–MS/MS—ranging from <5 to 151 nmol/L (<2.0–60.5 ng/mL) (median: 57.7 nmol/L; 23.1 ng/mL), with levels influenced by factors such as season, health status, and access to an outdoor enclosure [74]. Studies involving four primate species demonstrated 2–3-fold higher 25(OH)D_3_ concentrations following vitamin D_3_ administration compared with D_2_; in Macaca mulatta, D_3_ supplementation resulted in levels of 360 ± 60 nmol/L (144.2 ± 24.0 ng/mL), further highlighting interspecies differences in vitamin D metabolism [75]. In contrast, in three species of laboratory primates, mean 25(OH)D_3_ concentrations were 394.8 ± 43.5 nmol/L (158.2 ± 17.4 ng/mL), 154.8 ± 5.5 nmol/L (62.0 ± 2.2 ng/mL), and 164.8 ± 6.9 nmol/L (65.9 ± 2.8 ng/mL), with significant inter-individual variability [49].

These data confirm the utility of primates as models for vitamin D research but also demonstrate that 25(OH)D concentrations depend on species, environmental conditions, and individual factors, thereby limiting the applicability of universal reference ranges. Furthermore, Liptovszky et al. [76] emphasize the importance of standardizing sample collection and storage, assay methods, and result reporting, noting that methodological differences can further complicate comparisons across studies and species.

These findings may also provide a comparative context for understanding vitamin D status in humans, particularly in populations with high natural UVB exposure; however, the present review focuses on vertebrate animal species and does not aim to assess vitamin D status in human populations.

## 4. Vitamin D Status in Companion Animals

### 4.1. Cat (Felis catus)

Available evidence indicates that serum 25(OH)D concentrations in cats show considerable individual variability and are influenced primarily by dietary intake, age, and vitamin D supplementation (Table 2).

In cats, vitamin D status appears to be influenced primarily by dietary intake and age. In a study of 88 clinically healthy cats, serum 25(OH)D concentrations were determined using an ELISA, with a median concentration of 19.74 ng/mL. Cats fed homemade diets and those younger than six months had significantly lower concentrations, whereas no significant associations were found with sex, breed, reproductive status, or housing conditions [77].

The response to dietary 25(OH)D_3_ supplementation was further investigated in adult cats. Five cats received diets containing 4.9, 8.4, or 11.8 µg/kg of 25(OH)D_3_ for nine weeks, resulting in dose-dependent increases in plasma 25(OH)D from a baseline of 44 ± 4 ng/mL to 47 ± 5, 60 ± 7, and 65 ± 7 ng/mL, respectively. Vitamin D metabolites were quantified using HPLC and LC-MS/MS, supporting the efficacy of moderate 25(OH)D_3_ supplementation in increasing vitamin D status [78].

Reference concentrations have also been reported for different biological matrices. In three cats, mean total 25(OH)D concentrations measured by HPLC-MS/MS were 57.3 ± 8.7 nmol/L (22.9 ± 3.5 ng/mL) in plasma and 6.8 ± 2.2 nmol/kg in liver tissue [79]. However, the small sample size and differences between biological matrices limit direct comparisons, and no clear association was observed between plasma and liver 25(OH)D concentrations.

In conclusion, current evidence indicates that vitamin D status in cats is characterized by considerable individual variability and is influenced primarily by diet, age, and vitamin D intake. Moreover, appropriately selected 25(OH)D_3_ supplementation appears to be a safe and effective strategy for increasing circulating 25(OH)D concentrations.

### 4.2. Dogs (Canis lupus familiaris)

Available evidence indicates that dogs depend almost entirely on dietary vitamin D because cutaneous synthesis is negligible. Serum 25(OH)D concentrations are relatively high compared with many other mammalian species but are markedly influenced by disease status (Table 3).

In dogs, reduced serum 25(OH)D concentrations have been reported in association with various systemic diseases. In dogs with osteosarcoma, lymphoma, and mast cell tumors, mean serum 25(OH)D concentrations were 41.9 ± 20.6, 41.1 ± 14.4, and 44.9 ± 12.3 ng/mL, respectively, compared with 51.3 ± 16.7 ng/mL in healthy dogs, despite comparable dietary vitamin D intake. Plasma 25(OH)D and 24,25(OH)_2_D were measured using LC-MS. Serum 25(OH)D was associated with cancer type, 24,25(OH)_2_D, and ionized calcium concentrations, suggesting alterations in vitamin D metabolism in dogs with neoplastic disease [80].

A similar pattern was observed in dogs with hypercalcemia associated with lymphoma, primary hyperparathyroidism, or chronic renal failure. Serum 25(OH)D concentrations, measured using a protein-binding assay, were lower than in healthy dogs (165 nmol/L; 66 ng/mL), reaching 134 nmol/L (53.6 ng/mL), 116 nmol/L (46.4 ng/mL), and 84 nmol/L (33.6 ng/mL), respectively [81]. This finding suggests that systemic diseases may be associated with altered vitamin D metabolism and reduced circulating 25(OH)D concentrations.

Marked interspecies variation is also evident in dogs. Using HPLC-MS/MS, a multispecies study reported mean plasma total 25(OH)D concentrations of 129.8 ± 15.5 nmol/L (51.9 ± 6.2 ng/mL) in dogs and proposed a species-specific reference range of 56.1–179.1 nmol/L (22.4–71.6 ng/mL) [79]. The authors also emphasized the limited capacity for cutaneous vitamin D synthesis in dogs and their dependence on dietary vitamin D. Comparisons with other species should nevertheless consider differences in analytical methods, diet, environmental conditions, and study populations.

Taken together, these studies indicate that vitamin D status in dogs depends primarily on dietary vitamin D intake and the animal’s health status. Although relatively high serum 25(OH)D concentrations have been reported in dogs in some studies, significantly lower levels are consistently observed in animals with neoplastic diseases, chronic kidney disease, primary hyperparathyroidism, and lymphoma. These findings suggest that reduced serum 25(OH)D concentrations are associated with disease-related alterations in vitamin D metabolism and emphasize the importance of using species-specific reference ranges when assessing vitamin D status in dogs.

### 4.3. Domestic Rabbit (Oryctolagus cuniculus)

In domestic rabbits, serum 25(OH)D concentrations have been investigated in relation to PTH, bone density, diet, and environmental conditions. In a study of 139 rabbits, serum 25(OH)D concentrations measured by EIA ranged from 4.5 to 67.5 ng/mL, with a mean of 25.9 ± 11.7 ng/mL. A breakpoint in the relationship between 25(OH)D and PTH was identified at 17 ng/mL, which was proposed as a threshold for vitamin D deficiency, while a similar breakpoint for tibial cortical bone density was observed at 19 ng/mL [82].

In another study of 129 domestic rabbits, mean serum 25(OH)D concentration was 26.1 ng/mL, and dietary intake was significantly associated with vitamin D status, whereas outdoor access was not [83]. The highest concentrations were observed in rabbits receiving large amounts of hay together with at least 1 dL of commercial feed daily, suggesting that dietary intake may be more important than outdoor exposure under the conditions studied.

The relative contribution of direct UVB exposure and dietary vitamin D was further examined in 24 New Zealand White rabbits. Serum 25(OH)D_2_, 25(OH)D_3_, and total 25(OH)D were determined by LC-MS/MS. Direct UVB exposure increased 25(OH)D_3_ concentrations from 10.6 ± 2.2 ng/mL to 19.6 ± 3.7 ng/mL after two weeks and to 24.9 ng/mL after four weeks, whereas feeding UVB-irradiated hay resulted in higher 25(OH)D_2_ concentrations and total 25(OH)D levels of approximately 54–69 ng/mL [84]. These findings suggest that, under the conditions of this study, UVB irradiation of dietary hay may have a greater effect on vitamin D status than direct UVB exposure. However, the two approaches involved different exposure pathways and were not directly equivalent in terms of UVB dose.

In conclusion, current evidence indicates that vitamin D status in domestic rabbits depends primarily on dietary vitamin D intake, whereas the contribution of cutaneous synthesis appears to be limited. Serum 25(OH)D concentrations show considerable individual variation, and a concentration of approximately 17 ng/mL has been proposed as the threshold for vitamin D deficiency based on its association with increased PTH concentrations and reduced bone density. Comparisons among studies should, however, be interpreted with caution because differences in analytical methods (EIA versus LC-MS/MS) may influence the reported serum 25(OH)D concentrations.

A summary of studies evaluating vitamin D status in domestic rabbits is presented in Table 4.

## 5. Vitamin D Status in Livestock

### 5.1. Pig (Sus scrofa domesticus)

One of the earliest studies describing serum 25(OH)D_3_ concentrations in pigs was reported in [85]. Using high-performance liquid chromatography (HPLC), the authors measured plasma 25(OH)D_3_ concentrations in pigs kept outdoors and supplemented with vitamin D_3_ according to National Research Council recommendations. The mean plasma 25(OH)D_3_ concentration was 74.9 ± 21.2 ng/mL, providing an early reference point for vitamin D status in pigs. These findings provide an important historical reference for subsequent studies, illustrating changes in vitamin D status associated with the evolution of modern housing and feeding systems.

More recent data from pigs raised under commercial farming conditions showed a mean plasma total 25(OH)D concentration of 50.5 ± 6.1 nmol/L (20.2 ± 2.4 ng/mL), while the liver concentration was 7.8 ± 0.6 nmol/kg (3.1 ± 0.2 ng/kg). Total 25(OH)D was quantified using isotope-dilution liquid chromatography–tandem mass spectrometry (ID-LC-MS/MS). No clear correlation was observed between plasma and liver 25(OH)D concentrations, suggesting that these compartments may reflect vitamin D status differently. As the pigs were housed indoors and fed a commercial diet supplemented with vitamin D, both factors may have influenced the measured concentrations [79].

The effects of different forms of vitamin D supplementation have been investigated in pigs kept indoors without UVB exposure. In 160 prepubertal gilts receiving 5–50 µg/kg of vitamin D_3_ or 25(OH)D_3_ for 49 days, supplementation with 25(OH)D_3_ resulted in higher serum (11.4–67.1 ng/mL) and tissue (8.7–27.2 ng/mL) 25(OH)D_3_ concentrations than vitamin D_3_, with a strong correlation between serum and tissue concentrations [86].

In contrast, a study of 24 pigs receiving approximately 55 µg/day of vitamin D as vitamin D_3_, a mixture of vitamin D_3_ and 25(OH)D_3_, or 25(OH)D_3_ alone found no significant differences in plasma 25(OH)D_3_ concentrations among groups (18.1 ± 0.9, 21.4 ± 2.0, and 16.6 ± 1.3 ng/mL, respectively) [83]. These findings indicate that the relative effects of vitamin D_3_ and 25(OH)D_3_ supplementation may depend on the experimental conditions and the outcome assessed [87].

Large-scale observations also indicate an effect of housing conditions on vitamin D status. In a study including 1200 pigs housed indoors and 40 pigs with access to natural sunlight, all animals received commercial diets supplemented with vitamin D. Serum 25(OH)D concentrations were higher in outdoor pigs than in those kept indoors and increased with age in the indoor production system, from 13.75 ± 1.12 ng/mL in piglets to 45.42 ± 1.43 ng/mL in adult boars [88]. The authors noted that these concentrations were often below historical reference ranges, suggesting that current vitamin D supplementation strategies and reference intervals may require re-evaluation under modern production conditions.

The timing of sample collection may also affect measured vitamin D concentrations. In eight pigs fed a soybean meal- and corn-based diet supplemented with a commercial vitamin–mineral premix, serum 25(OH)D_3_ was measured by LC-MS/MS before and after slaughter. Mean concentrations were 21.02 ± 1.81 ng/mL in live animals and 19.34 ± 3.11 ng/mL in post-mortem samples, with significantly lower values after slaughter [89]. These findings suggest that post-mortem changes may influence vitamin D measurements and support the use of samples collected from live animals when assessing vitamin D status.

The available evidence indicates that vitamin D status in pigs is influenced primarily by housing conditions, UVB exposure, and vitamin D supplementation. Within the studies reviewed, outdoor-reared pigs generally had higher serum 25(OH)D_3_ concentrations than pigs maintained under intensive indoor production systems, although the populations differed in several management and nutritional factors. In addition, supplementation with 25(OH)D_3_ appears to increase serum and tissue 25(OH)D_3_ concentrations more effectively than vitamin D_3_, highlighting the importance of appropriate vitamin D supplementation in pigs raised without natural UVB exposure. A summary of studies evaluating vitamin D status in pigs is presented in Table 5.

### 5.2. Cattle (Bos taurus)

One of the earliest studies to determine serum 25(OH)D_3_ concentrations in cattle using high-performance liquid chromatography (HPLC) included adult animals kept outdoors with access to sheltered areas and supplemented with vitamin D according to National Research Council recommendations. Blood samples were collected in October, and the mean plasma 25(OH)D_3_ concentration was 36.9 ± 7.1 ng/mL. The HPLC method allowed simultaneous quantification of multiple vitamin D metabolites and represented an important methodological advance in studies of vitamin D metabolism in livestock [85]. Although historical, these findings provide a useful reference for comparison with more recent studies conducted under different environmental and nutritional conditions.

Housing conditions and vitamin D supplementation also appear to influence vitamin D status in cattle. In heifers studied during winter, serum 25(OH)D concentrations were similar in indoor- and outdoor-housed animals (approximately 19 ng/mL), whereas in summer, pasture-grazed heifers had significantly higher concentrations than those kept indoors (approximately 49 vs. 21 ng/mL). In a second experiment, winter supplementation with vitamin D_3_ increased serum 25(OH)D concentrations, with the highest values observed after a single intramuscular injection of 1,000,000 IU (27 ± 1.8 ng/mL) [90]. These findings indicate that seasonal UVB exposure is an important determinant of vitamin D status in cattle, while supplementation may partially compensate for reduced winter exposure.

Seasonal variation was also observed in 195 beef calves raised in the central United States. The calves grazed on pasture until 5–6 months of age and were subsequently transferred to a finishing facility where they received a vitamin D-supplemented diet. Serum 25(OH)D concentrations were determined by radioimmunoassay (RIA), with the lowest values observed during winter finishing (15.2 ± 1.6 and 16.7 ± 1.5 ng/mL) and the highest in late summer and early autumn (46.6–63.8 ng/mL) [91]. These results further illustrate the importance of seasonal changes in UVB exposure and suggest that dietary vitamin D supplementation may not fully compensate for reduced endogenous synthesis during winter housing.

More recent multispecies data obtained using a standardized analytical approach provide additional information on vitamin D status in cattle. In pasture-raised animals, mean total 25(OH)D concentrations measured by ID-LC-MS/MS were 98.3 ± 6.4 nmol/L (39.3 ± 2.6 ng/mL) in plasma and 2.1 ± 0.6 nmol/kg (0.84 ± 0.24 ng/kg) in liver tissue [79]. The use of the same analytical method across species facilitates comparison, although differences in biological matrices, environmental conditions, and study populations should still be considered when interpreting interspecies variation.

In cattle, vitamin D status also depends on skin exposure to light. In a study involving 16 Danish Holstein dairy cows deprived of vitamin D_3_ supplementation for six months, the animals were fed a diet without added vitamin D_3_ and spent five hours daily on pasture. 25(OH)D_3_ concentrations were determined using HPLC with UV detection. After 28 days, the levels were 28.6 ± 3.1 ng/mL for cows with no covering, 23.2 ± 1.5 ng/mL with an udder cover, 8.9 ± 1.8 ng/mL with a blanket, and 6.0 ± 0.5 ng/mL with both a blanket and an udder cover. The results indicate that vitamin D_3_ synthesis occurs across the entire skin surface, including under the coat, and that limiting exposure via a blanket reduces 25(OH)D_3_ concentrations [92].

The available studies indicate that vitamin D status in cattle depends primarily on seasonal UVB exposure and, to a lesser extent, on dietary supplementation. The highest serum 25(OH)D concentrations are consistently observed in pasture-grazed cattle during summer, whereas marked declines occur during winter, particularly in animals housed indoors. Although vitamin D supplementation increases serum 25(OH)D concentrations during periods of limited UVB exposure, it does not always maintain concentrations comparable to those observed during the grazing season. The reviewed studies also demonstrate interspecies differences in vitamin D status and the absence of a clear correlation between plasma and liver 25(OH)D concentrations, underscoring the importance of using species-specific reference values when evaluating vitamin D status in cattle. The main findings of studies evaluating vitamin D status in cattle are summarized in Table 6.

### 5.3. Horses (Equus caballus)

Seasonal variation in vitamin D status has been documented in horses. In 142 racehorses (Standardbred and Finnhorse) aged 3–15 years, serum 25(OH)D concentrations measured by HPLC were 1.90 ± 0.23 ng/mL in winter and 2.43 ± 0.09 ng/mL in summer. During winter, the horses were fed hay and oats, whereas in summer they received fresh grass, hay, oats, and a vitamin–mineral supplement. The seasonal increase in serum 25(OH)D concentrations suggests a greater risk of vitamin D deficiency during winter in horses kept in Northern Europe [93]. Although the study used an older analytical method, it provides a useful historical reference for seasonal variation in vitamin D status.

The influence of season and breed was also examined in 90 healthy horses (Turkmen, Thoroughbred, and crossbreeds) kept in stables in northern Iran. The horses had no access to pasture but were exposed to sunlight for several hours daily and received alfalfa hay throughout the year. Serum 25(OH)D concentrations measured by ELISA had a median value of 17.42 ng/mL (IQR: 9.82–30.85 ng/mL) and were significantly lower in autumn than in spring (15.83 vs. 18.02 ng/mL) and in Turkmen horses than in Turkmen–Thoroughbred crossbreeds [94]. These findings further support the importance of season in determining vitamin D status, although the relative contributions of sunlight exposure and dietary vitamin D remain uncertain.

Age-related differences have also been reported in horses and ponies. In 91 healthy animals from Thailand and the United States with regular access to paddocks and natural sunlight, serum 25(OH)D_3_ concentrations measured by EIA were lower in foals than in yearlings and adults in both countries. In Thailand, adult Thoroughbreds also had higher concentrations than ponies (18.4–30.5 vs. 5.7–13.1 ng/mL), whereas no significant differences were observed between animals from Thailand and the United States [95]. These results indicate that age and breed may contribute to variation in vitamin D status, while comparisons across studies remain limited by differences in analytical methods and study conditions.

In horses, vitamin D status can depend on both environmental conditions and dietary sources. A study conducted in New Zealand on 21 pasture-kept horses found no significant differences in 25(OH)D_2_ and 25(OH)D_3_ concentrations between horses blanketed for 13 months and unblanketed horses, even though blanketing limited the skin surface area exposed to light. Metabolite concentrations were determined using the ID-LC-MS/MS method. Notably, 25(OH)D_3_ was undetectable in 78% of the samples, and 25(OH)D_2_ was the predominant form of 25(OH)D. Its concentrations were higher in spring and summer than in autumn and winter, indicating the importance of seasonality, while the predominance of 25(OH)D_2_ may suggest a significant contribution of dietary sources to maintaining vitamin D status in horses [96].

The available studies indicate that vitamin D status in horses is influenced by several factors, of which season appears to be the most consistent determinant. The effects of age and breed have not been consistently demonstrated across studies. Comparisons between studies are further complicated by differences in analytical methods, as well as variations in housing and feeding practices.

Table 7 summarizes the available studies on vitamin D status in horses, including study design, analytical methods, and reported serum 25(OH)D concentrations.

### 5.4. Sheep (Ovis aries)

In adult sheep kept outdoors with access to sheltered areas, mean plasma 25(OH)D concentration was 12.9 ± 5.3 ng/mL. The animals received vitamin D_3_ supplementation and diets meeting National Research Council recommendations for calcium, phosphorus, and vitamin D. Despite continuous sunlight exposure and supplementation, vitamin D_2_ and 25(OH)D_2_ were the predominant circulating forms, suggesting that dietary vitamin D_2_ may represent an important source of vitamin D in sheep. The limited contribution of cutaneous vitamin D_3_ synthesis may be related to the thick wool coat and lanolin, which can reduce UVB penetration [85].

Pregnancy status may also influence vitamin D concentrations. In 200 pasture-raised Romney sheep in New Zealand, mean total serum 25(OH)D concentration was 97.9 ± 1.8 nmol/L (39.16 ± 0.72 ng/mL). Concentrations were similar in ewes carrying singletons and twins but were significantly lower in ewes carrying triplets (33.20 ± 1.80 ng/mL), possibly reflecting the greater metabolic demands of multiple pregnancies. However, the observational design does not allow causal conclusions [97].

Breed-related differences in vitamin D status were observed in 259 Scottish Blackface and Lleyn sheep maintained under extensive grazing conditions. Mean total serum 25(OH)D concentrations ranged from 15.2 to 17.7 ng/mL, with most animals (91.9%) showing concentrations between 10 and 30 ng/mL. Lleyn sheep had significantly higher 25(OH)D_3_ concentrations than both Scottish Blackface populations. Higher serum 25(OH)D and 25(OH)D_3_ concentrations were also positively associated with lamb birth weight, although no association was found with the number of lambs born or reared [98].

A multispecies study using the same isotope-dilution LC-MS/MS method reported a mean plasma total 25(OH)D concentration of 27.5 ± 1.9 nmol/L (11.0 ± 0.8 ng/mL) in four pasture-raised sheep that did not receive additional vitamin D supplementation [79]. Although these data provide a useful reference for comparison with other species, they should be interpreted cautiously because of the very small sample size and the potential influence of species-specific, environmental, and methodological factors.

The available evidence demonstrates considerable variation in serum 25(OH)D concentrations in sheep, ranging from approximately 11.0 to 40.8 ng/mL, depending on breed, physiological status, husbandry system, diet, and season. Higher concentrations have been reported in pasture-raised sheep in New Zealand, whereas lower values were observed in sheep maintained under extensive grazing conditions in Scotland and in small reference populations. Vitamin D status also appears to be influenced by breed and physiological status, with Lleyn sheep exhibiting higher 25(OH)D_3_ concentrations than Scottish Blackface sheep and ewes carrying triplets showing lower 25(OH)D concentrations than those carrying singletons or twins. Available evidence further indicates that vitamin D_2_ and 25(OH)D_2_ remain the predominant circulating forms in sheep, reflecting species-specific characteristics of vitamin D metabolism. Consequently, the interpretation of serum 25(OH)D concentrations in sheep should take into account both biological and environmental factors.

The available studies evaluating vitamin D status in sheep are summarized in Table 8.

### 5.5. Poultry–Chicken (Gallus gallus domesticus) and Turkey (Meleagris gallopavo)

Available data on baseline serum 25(OH)D concentrations in chickens and turkeys remain limited. Moreover, most published studies have been conducted under conditions involving vitamin D supplementation or other nutritional interventions, making it difficult to assess the natural vitamin D status of these species.

Under indoor conditions without direct sunlight, circulating vitamin D metabolites reflected primarily dietary vitamin D_3_ intake. In chickens and turkeys receiving diets supplemented according to National Research Council recommendations, mean plasma 25(OH)D_3_ concentrations were 25.3 ± 3.9 ng/mL and 18.2 ± 7.9 ng/mL, respectively. Vitamin D_3_ and 25(OH)D_3_ were the predominant circulating forms of vitamin D and 25(OH)D, respectively. These findings illustrate the influence of both species-specific metabolism and dietary vitamin D intake on circulating vitamin D metabolite profiles in the absence of direct UVB exposure [85].

The form of vitamin D supplementation also affects circulating 25(OH)D_3_ concentrations in broiler chickens. When vitamin D_3_ and 25(OH)D_3_ were provided at equal weight-based doses of 69 µg/kg of feed, the mean serum 25(OH)D_3_ concentrations were 13.3 ± 4.3 ng/mL and 42.5 ± 18.0 ng/mL, respectively, indicating an approximately 3.2-fold higher concentration following 25(OH)D_3_ supplementation [99]. This greater response is consistent with the position of 25(OH)D_3_ one step further along the vitamin D metabolic pathway, thereby bypassing hepatic 25-hydroxylation. Serum concentrations were also higher than tissue concentrations and were strongly correlated with tissue 25(OH)D_3_ content, supporting serum 25(OH)D_3_ as an indicator of vitamin D status.

A similar, although less pronounced, effect of 25(OH)D_3_ supplementation was observed in another study of broiler chickens. In birds receiving the control diet, which contained 2760 IU/kg feed of vitamin D_3_, mean serum 25(OH)D concentrations were 25.11 ng/mL on day 21 and 23.77 ng/mL on day 42, whereas supplementation with 69 µg/kg feed of 25(OH)D_3_ increased these values to 31.49 and 28.81 ng/mL, respectively [100].

In summary, the available evidence indicates that baseline vitamin D status in chickens and turkeys remains insufficiently characterized because most studies have been conducted under conditions of dietary vitamin D supplementation. In intensively reared poultry, reported serum 25(OH)D_3_ concentrations generally range from approximately 13 to 42 ng/mL, with consistently higher values following 25(OH)D_3_ supplementation than after vitamin D_3_ administration. These findings emphasize the predominant role of dietary vitamin D intake in determining vitamin D status in poultry and highlight the limited availability of reference data obtained under non-supplemented conditions.

The available studies evaluating vitamin D status in chickens and turkeys are summarized in Table 9.

### 5.6. Fish

In fish, dietary intake is the primary source of vitamin D_3_. In the liver, cholecalciferol is hydroxylated to 25(OH)D_3_, the major circulating and storage form of vitamin D and the principal biomarker of vitamin D status. Subsequently, 25(OH)D_3_ is converted to the biologically active metabolite, 1,25(OH)_2_D_3_, whose circulating concentration in fish has been reported to be 10–55 times higher than in mammals [101].

In rainbow trout (*Oncorhynchus mykiss*), dietary 25(OH)D_3_ supplementation produced a clear dose-dependent increase in plasma 25(OH)D_3_ concentrations. Fish received a diet containing the recommended level of vitamin D_3_ supplemented with 25(OH)D_3_ at 69.8, 687, or 6854.3 µg/kg feed for 91 days. Plasma 25(OH)D_3_ was measured using ultra-high-performance liquid chromatography–tandem mass spectrometry (UHPLC-MS/MS). In control fish, concentrations remained below the detection limit (<1.0 ng/mL), whereas supplementation increased them to 0.38 ± 0.20, 8.5 ± 2.3, and 95.5 ± 2.3 ng/mL, respectively. The higher supplementation levels were also associated with improved growth performance and feed efficiency [102].

A similar dose-dependent response was observed when cholecalciferol and 25(OH)D_3_ were compared in 480 rainbow trout reared under indoor conditions. Fish received diets containing vitamin D_3_ or 25(OH)D_3_ at 200, 400, or 800 µg/kg feed for 84 days, and vitamin D metabolites in plasma, muscle, and liver were quantified by HPLC. Plasma 25(OH)D_3_ concentrations increased from approximately 0–1 ng/mL in fish receiving no 25(OH)D_3_ to 90–100 ng/mL at the highest supplementation level. The study also demonstrated limited conversion of vitamin D_3_ to 25(OH)D_3_ and confirmed the absence of endogenous vitamin D_3_ synthesis under indoor conditions. Thus, direct supplementation with 25(OH)D_3_ was more effective than vitamin D_3_ in increasing circulating and tissue 25(OH)D_3_ concentrations [103].

Most studies have focused on vitamin D concentrations in tissues and plasma 25(OH)D concentrations in farmed fish. In contrast, data comparing vitamin D status between farmed and wild fish remain scarce. Nevertheless, differences in vitamin D content were observed between the tissues of farmed and wild fish. For example, differences in tissue vitamin D content have been reported between farmed and wild salmon, with farmed salmon containing approximately 25% of the vitamin D content reported in wild salmon [104]. However, this observation concerns tissue vitamin D content rather than circulating 25(OH)D and therefore should not be interpreted directly as evidence of differences in systemic vitamin D status. Considering the growing consumption of fish and the increasing availability of products originating from both aquaculture and natural ecosystems [105,106], comparative studies evaluating serum or plasma 25(OH)D concentrations in these populations would provide valuable insights into species-specific vitamin D status and its environmental determinants.

The available studies evaluating vitamin D status in fish are summarized in Table 10.

### 5.7. Reptiles

In reptiles, vitamin D status is influenced by several factors, including UVB exposure, dietary vitamin D_3_ intake, radiation intensity, and species-specific ecological adaptations. Studies on anoles have demonstrated an adaptation of cutaneous vitamin D_3_ synthesis to UVB availability and dietary vitamin D_3_ intake [107], while research on panther chameleons has shown that UVB radiation intensity can affect vitamin D_3_ content in eggs and hatching success [108]. In green sea turtles, 25(OH)D concentrations were higher in animals kept outdoors compared with those kept indoors and were positively correlated with calcium levels [109].

In bearded dragons (*Pogona vitticeps*), vitamin D status varied according to the characteristics of the light source. After 11 months of exposure to UVB radiation, UVB-emitting LED light, or light without UVB, mean plasma 25(OH)D_3_ concentrations reached 74.8 ng/mL in the LED group, 62.3 ng/mL in the UVBN group, and 54.3 ng/mL in the UVB group. Plasma 25(OH)D_3_ was determined using LC-MS/MS, and the results indicate that the type of artificial light can influence vitamin D status [110].

Cutaneous vitamin D_3_ synthesis also occurs in the nocturnal leopard gecko (*Eublepharis macularius*). Following UVB exposure, plasma 25(OH)D_3_ concentrations increased from 15.2 ± 3.2 to 24.4 ± 8.0 ng/mL. Concentrations were determined using isotope-dilution on-line solid-phase extraction liquid chromatography–tandem mass spectrometry (ID-XLC-MS/MS), demonstrating that UVB can stimulate vitamin D_3_ synthesis even in a nocturnal reptile [111].

Considerable variation in vitamin D status has also been reported among wild Galápagos iguanas (*Conolophus marthae*, *C. subcristatus*, and *C. pallidus*). Total 25(OH)D concentrations ranged from 17.3 to 357 ng/mL and were measured using a chemiluminescence immunoassay (CLIA), while additional HPLC analysis showed that vitamin D_3_ accounted for 95–98% of total vitamin D. The substantial interspecific variation may reflect differences in species-specific physiology as well as ecological and individual factors [112].

The available studies indicate that reported 25(OH)D concentrations in reptiles are influenced by multiple factors, including UVB exposure, dietary vitamin D_3_ intake, radiation intensity, and species-specific ecological characteristics. The observed variation among species should therefore be interpreted cautiously, particularly because studies differed in analytical methods and experimental or environmental conditions, limiting the establishment of universal 25(OH)D reference values.

The available studies evaluating vitamin D status in reptiles are summarized in Table 11.

## 6. Vitamin D Status in Wildlife

Determining serum 25(OH)D concentrations in free-living animals is important not only for understanding species-specific physiology but also for assessing the health status of wild populations [113]. Vitamin D plays a central role in calcium and phosphate homeostasis, bone mineralization, and immune function [31,113,114]. Increasing evidence also suggests that vitamin D status influences susceptibility to infectious diseases and modulates immune responses [31,115].

In wild animals, particularly species serving as reservoirs of infectious pathogens, assessment of vitamin D status may complement population health monitoring and provide valuable information for disease surveillance and epidemiological studies, as demonstrated in investigations of tuberculosis in red deer [116]. In addition, monitoring the health status of game species is important from a food safety perspective because meat from wild animals is consumed by humans, making its quality and safety relevant to public health [117,118,119].

### 6.1. Wild Boar (Sus scrofa)

In a study involving 296 wild boars from 27 hunting grounds in west-central Spain, the mean serum 25(OH)D concentration was 20.69 ± 24.99 ng/mL. Animals from 19 hunting grounds had year-round access to pelleted feed containing vitamin D_3_ (2000 IU/kg), whereas the others subsisted solely on natural forage. Serum 25(OH)D concentrations were determined using the ELISA method. No significant differences related to sex or age were observed; however, a distinct seasonal effect was evident, with the lowest values recorded in winter (11.2 ng/mL) and the highest in summer (34.8 ng/mL). Both sunlight exposure and vitamin D_3_ supplementation were positively associated with serum 25(OH)D concentrations [44].

The effect of vitamin D_3_ supplementation was also evaluated in 20 wild boars from four fenced hunting grounds in west-central Spain. Ten animals received feed supplemented with vitamin D_3_ (approximately 2000 IU/day), while the others received no supplementation. Serum concentrations of 25(OH)D_3_ and 25(OH)D_2_ were determined using HPLC. In the supplemented wild boars, the 25(OH)D_3_ concentration was significantly higher than in the non-supplemented animals (14.39 vs. 6.15 nmol/L), whereas no differences were observed in 25(OH)D_2_ concentrations [116]. Higher 25(OH)D_3_ concentrations were also observed in animals with localized tuberculosis compared with wild boars with the generalized form, which may indicate a link between vitamin D status and the course of *Mycobacterium bovis* infection. The original publication reports serum 25(OH)D concentrations in nmol/mL in Table 1; however, the text of the article reports the values in nmol/L. Therefore, the unit “nmol/mL” in Table 1 is most likely a typographical error.

In a group of 56 wild boars harvested during the 2016–2017 and 2017–2018 hunting seasons in northeastern Spain, serum 25(OH)D_3_ concentrations were determined using LC-MS/MS. Concentrations were higher in adults than in juveniles, measuring 7.08 ± 0.86 ng/mL in adult females, 4.32 ± 1.17 ng/mL in adult males, 2.04 ± 1.65 ng/mL in juvenile females, and 2.56 ± 1.22 ng/mL in juvenile males [89]. The study also revealed lower vitamin D_3_ concentrations in post-mortem samples compared with samples collected from live animals, and provided the first reference values for vitamins A, D, and E in wild boars.

Another study evaluated total 25(OH)D concentrations in the livers of six wild boars from New Zealand that received no vitamin D supplementation and subsisted solely on natural forage. Analyses were performed using the ID-LC-MS/MS method, and the mean hepatic concentration was 2.5 ± 0.8 nmol/kg, compared with 7.8 ± 0.6 nmol/kg in domestic pigs examined in the same study [79]. This difference may stem in part from variations in diet, housing conditions, and environmental exposure—specifically, vitamin D supplementation in commercial pig diets. The results thus indicate that diet can significantly influence hepatic vitamin D stores.

The available evidence indicates that vitamin D status in wild boars is influenced primarily by season, sunlight exposure, and diet. Higher serum 25(OH)D concentrations have been reported in animals receiving vitamin D_3_ supplementation and in adults, whereas lower 25(OH)D concentrations have been reported in some wild boar populations than in commercially raised pigs; however, these differences cannot be attributed solely to species-specific physiology because the populations differed in diet, supplementation, husbandry, and environmental exposure. Moreover, higher serum 25(OH)D_3_ concentrations were associated with a milder course of *Mycobacterium bovis* infection, suggesting a potential role of vitamin D in the immune response of this species.

The available studies evaluating vitamin D status in wild boars are summarized in Table 12.

### 6.2. Red Deer (Cervus elaphus)

Total 25(OH)D concentrations in plasma and liver were determined using the ID-LC-MS/MS method in four free-ranging red deer (*Cervus elaphus*) from New Zealand that had not received vitamin D supplementation. The mean total 25(OH)D concentration was 12.8 ± 1.1 nmol/L (5.1 ± 0.4 ng/mL) in plasma and 5.4 ± 0.2 nmol/kg in the liver [79]. The study provided reference values for vitamin D status in deer; however, interspecies comparisons should be interpreted with caution due to the potential influence of environmental conditions and methodological factors.

The effect of vitamin D_3_ supplementation was also evaluated in 20 deer from four fenced hunting estates in central-western Spain. Ten animals received feed supplemented with vitamin D_3_ (approximately 8000 IU/day), while the others received unsupplemented feed. Serum concentrations of 25(OH)D_3_ and 25(OH)D_2_ were determined using HPLC. In the supplemented deer, the 25(OH)D_3_ concentration was significantly higher than in the non-supplemented animals (91.62 vs. 7.65 nmol/L), whereas no significant differences were found in 25(OH)D_2_ concentrations [116]. Higher 25(OH)D_3_ concentrations were also observed in animals with localized tuberculosis compared with deer with the generalized form, which may indicate a link between vitamin D status and the course of *Mycobacterium bovis* infection. It should be noted, however, that the table in the publication lists the unit as nmol/mL, whereas the values in the text are presented in nmol/L, most likely indicating a typographical error in the table.

The available evidence indicates that vitamin D status in red deer is influenced by dietary vitamin D_3_ supplementation and is associated with the severity of *Mycobacterium bovis* infection. Supplementation markedly increased serum 25(OH)D_3_ concentrations, while higher vitamin D status was associated with a more localized form of tuberculosis, suggesting a potential role of vitamin D in the immune response of this species. The available studies evaluating vitamin D status in red deer are summarized in Table 13.

### 6.3. Wild Rabbit (Oryctolagus cuniculus)

To date, only one study has evaluated serum 25(OH)D concentrations in wild rabbits. In animals from southern Finland, the mean serum 25(OH)D concentration was 3.3 ng/mL (range: 0.3–7.1 ng/mL), with no significant differences according to season or sex [43]. These findings highlight the limited knowledge of vitamin D status in wild rabbit populations and the need for further studies to establish reference values and identify the factors influencing vitamin D status in this species. The available study evaluating vitamin D status in wild rabbits is summarized in Table 14.

## 7. Comparison Analysis of Vitamin D Status in Domestic and Wild Vertebrates

### 7.1. Pig (Sus scrofa domesticus) vs. Wild Boar (Sus scrofa)

Comparison of domestic pigs and wild boars illustrates the potential influence of environmental, nutritional, and management conditions on reported 25(OH)D concentrations. However, because these populations differ simultaneously in genetics, diet, UVB exposure, husbandry, and other factors, the available observational data do not allow the relative contributions of species-specific physiology and environmental conditions to be determined. Outdoor-reared pigs exhibited the highest reported serum 25(OH)D concentrations (74.9 ± 21.2 ng/mL) [85], whereas pigs raised under modern indoor production systems showed concentrations comparable to those observed in wild boars, generally ranging from 16.6 to 21.4 ng/mL [79,87,89]. In free-ranging wild boars, mean serum 25(OH)D concentration was 20.69 ± 24.99 ng/mL, with pronounced seasonal variation from 11.2 ng/mL in winter to 34.8 ng/mL in summer [44], reflecting changes in sunlight exposure. Differences were also observed in hepatic vitamin D stores, with pigs exhibiting more than threefold higher liver 25(OH)D concentrations than wild boars [79], most likely reflecting continuous dietary supplementation in commercial production systems. Experimental studies further demonstrated that supplementation with 25(OH)D_3_ increased circulating vitamin D concentrations more efficiently than vitamin D_3_ alone [86]. These findings indicate that husbandry practices, UVB exposure, and dietary vitamin D intake may substantially influence reported vitamin D status; however, the available comparisons do not allow their relative contribution to be separated from species-specific physiological differences.

### 7.2. Cattle (Bos taurus) vs. Red Deer (Cervus elaphus)

A similar pattern was observed in the available comparisons between cattle and red deer, although the populations differed in environmental exposure, diet, management, and other characteristics that may influence vitamin D status. In cattle, serum 25(OH)D concentrations increased during the grazing season and reached 36.9–63.8 ng/mL [79,85,91], whereas winter housing resulted in concentrations of approximately 15–21 ng/mL despite dietary supplementation [90,91].

Free-ranging red deer exhibited substantially lower circulating 25(OH)D concentrations (5.1 ± 0.4 ng/mL) than cattle [79]. However, vitamin D_3_ supplementation increased serum concentrations to 36.65 ng/mL, approaching those reported in domestic cattle [116]. Interestingly, hepatic 25(OH)D concentrations were higher in deer than in cattle, which may suggest differences in tissue distribution or vitamin D metabolism; however, the available data do not allow these possibilities to be distinguished from differences in environmental and nutritional exposure. Overall, these findings highlight the important contribution of seasonal UVB exposure and dietary vitamin D supply to vitamin D status in the studied ruminant populations. However, the relative contribution of these factors versus species-specific physiology cannot be determined from the available observational comparisons.

### 7.3. Domestic Rabbit (Oryctolagus cuniculus) vs. Wild Rabbit (Oryctolagus cuniculus)

Unlike pigs and ruminants, rabbits exhibit a distinct pattern of vitamin D metabolism in which dietary intake appears to be considerably more important than cutaneous synthesis. Domestic rabbits maintained serum 25(OH)D concentrations of approximately 26 ng/mL despite limited effects of outdoor access [82,83]. Experimental studies further demonstrated that feeding UVB-irradiated hay increased serum 25(OH)D concentrations more effectively than direct UVB exposure [43], highlighting the predominant contribution of dietary vitamin D.

In contrast, wild rabbits in the available study showed lower mean serum 25(OH)D concentrations (mean 3.3 ng/mL) than those reported in domestic rabbits; however, the two populations differed in diet, housing, environmental exposure, and study conditions, limiting direct attribution of this difference to domestication or physiology [43]. These findings, together with the experimental data in domestic rabbits, suggest that dietary vitamin D availability is an important determinant of vitamin D status in rabbits, whereas the contribution of endogenous synthesis appears to be limited under the conditions studied.

## 8. Limitations

This review has several limitations that should be considered when interpreting differences in 25(OH)D concentrations across species. Direct interspecies comparisons are constrained by variations in analytical methods, their standardization, and the biological matrices analyzed. These factors may influence the measured 25(OH)D concentrations, particularly when analytical methods have not been validated for a specific species or biological material [120]. Another important consideration is vitamin D-binding protein (VDBP), as the binding properties of vitamin D metabolites vary among species. These differences may affect the proportion of free 25(OH)D and, consequently, the interpretation of total 25(OH)D concentration as an indicator of vitamin D status [28,120]. In addition, the available data are unevenly distributed across species, with markedly less information for wild animals than for domestic, livestock, laboratory, or captive animals. Finally, 25(OH)D concentrations should be interpreted within a broader physiological context, given the close relationships between vitamin D metabolism, calcium–phosphate balance, and mineral homeostasis [121,122].

## 9. Conclusions

The available evidence demonstrates substantial interspecies variation in vitamin D metabolism and serum 25(OH)D concentrations among vertebrates. These differences reflect the combined effects of species-specific physiology, dietary vitamin D intake, UVB exposure, husbandry practices, and environmental conditions. Across the studies reviewed, UVB exposure and diet emerged as the principal determinants of vitamin D status, although their relative importance varied considerably among species. In pigs, cattle, and wild boars, both sunlight exposure and dietary supplementation strongly influenced circulating 25(OH)D concentrations, whereas in rabbits, dietary intake appeared to play a dominant role. Moreover, supplementation with 25(OH)D_3_ consistently proved more effective than cholecalciferol in increasing circulating 25(OH)D concentrations. Importantly, numerical differences in reported 25(OH)D concentrations should be interpreted cautiously because analytical methods, biological matrices, sampling strategies, and reporting conventions varied substantially among studies. Consequently, observed differences between species cannot always be attributed to physiological differences alone.

This review also highlights several important limitations of the current literature. Differences in analytical methods, sampling strategies, and reference values complicate comparisons between studies and species. In addition, information on vitamin D status in free-ranging wildlife remains scarce, particularly for species of ecological and veterinary importance. Future research should therefore prioritize the standardized measurement of serum 25(OH)D using validated analytical methods, the establishment of species-specific reference intervals, and investigation of the physiological and immunological roles of vitamin D under both natural and managed conditions.

## Figures and Tables

**Table 1:** General characteristics of vitamin D sources in different animal groups.

Animal Group	Predominant Source of Vitamin D	Importance of UVB Exposure	Importance of Dietary Intake	Seasonal Variation	Main Influencing Factors	Reference
Companion animals	Diet	Low	High	Low	Feed composition, indoor lifestyle	[11,32]
Livestock animals	Mixed	Moderate	Moderate to high	Moderate	Husbandry system	[31]
Wild animals	Mixed, with stronger environmental dependence	Moderate to high	Moderate	High	Environment, climate, seasonal sunlight exposure	[43,44]

**Table 2:** Summary of studies evaluating vitamin D status in cats.

Assay	25(OH)D Metabolites (ng/mL)	Diet/Vitamin D Source	Key Finding	Reference
ELISA	Median: 19.74	Commercial, mixed, or homemade diets	Lower 25(OH)D concentrations in kittens (<6 months) and cats fed homemade diets	[77]
HPLC, LC-MS/MS	Mean ± SD: Baseline: 44 ± 4; 47 ± 5, 60 ± 7, and 65 ± 7 after supplementation	25(OH)D_3_ supplementation (4.9, 8.4, or 11.8 µg/kg diet)	Dose-dependent increase in 25(OH)D concentrations following supplementation	[78]
HPLC-MS/MS	Mean total ± SD: 22.9 ± 3.5	Not reported	Reference plasma 25(OH)D concentrations established	[79]

**Table 3:** Summary of studies evaluating vitamin D status in dogs.

Assay	25(OH)D Metabolites (ng/mL)	Diet/Vitamin D Source	Key Finding	Reference
LC-MS	Mean ± SD: Healthy: 51.3 ± 16.7; Osteosarcoma: 41.9 ± 20.6; Lymphoma: 41.1 ± 14.4; Mast cell tumor: 44.9 ± 12.3	Comparable dietary vitamin D intake	Lower 25(OH)D concentrations in dogs with cancer than in healthy dogs	[80]
Protein-binding assay	Median: Healthy: 66.0; Lymphoma: 53.6; Primary hyperparathyroidism: 46.4; Chronic kidney disease: 33.6	Not reported	Lower 25(OH)D concentrations in dogs with systemic diseases	[81]
ID-LC-MS/MS	Mean ± SD: 51.9 ± 6.2; Reference range: 22.4–71.6	Dietary vitamin D	Species-specific reference range for dogs; vitamin D depends on dietary intake	[79]

**Table 4:** Summary of studies evaluating vitamin D status in rabbits.

Assay	25(OH)D Metabolites (ng/mL)	Diet/Vitamin D Source	Key Finding	Reference
EIA	Mean: 26.1 (range: 4.5–67.5)	Hay and commercial feed	Diet, but not outdoor access, significantly affected 25(OH)D concentrations	[82]
EIA	Mean ± SD: 25.9 ± 11.7 (range: 4.5–67.5)	Standard diet	Vitamin D deficiency threshold estimated at 17 ng/mL	[83]
LC-MS/MS	Control: ~38–40; Direct UVB: ~44–46; UVB-irradiated hay: ~54–56	UVB-irradiated hay vs. direct UVB exposure	UVB-irradiated hay increased total 25(OH)D more effectively than direct UVB exposure	[84]

**Table 5:** Summary of studies evaluating vitamin D status in pigs.

Assay	25(OH)D Metabolites (ng/mL)	Diet/Vitamin D Source	Key Finding	Reference
HPLC	Mean ± SD: 74.9 ± 21.2	Vitamin D_3_ supplementation (National Research Council-NRC recommendations)	Higher 25(OH)D_3_ concentrations reported in outdoor pigs than in indoor pigs in the same study	[85]
HPLC	Mean ± SD: 18.1 ± 0.9 (vitamin D_3_); 21.4 ± 2.0 (vitamin D_3_ + 25(OH)D_3_); 16.6 ± 1.3 (25(OH)D_3_)	Vitamin D_3_ and/or 25(OH)D_3_ supplementation	Vitamin D_3_ and 25(OH)D_3_ showed comparable biological activity	[87]
HPLC-MS/MS (isotope dilution)	Mean total ± SD: 20.2 ± 2.4	Commercial diet	Reference plasma 25(OH)D concentrations established	[79]
Heartland Assays LLC	Mean ± SD: 13.8 ± 1.1 (piglets) to 45.4 ± 1.4 (boars)	Commercial diet with vitamin D	Outdoor pigs had higher 25(OH)D concentrations than indoor pigs	[88]
HPLC	Range: 11.4–67.1	Vitamin D_3_ or 25(OH)D_3_ supplementation	25(OH)D_3_ supplementation was more effective than vitamin D_3_	[86]
LC-MS/MS	Mean ± SD: 21.0 ± 1.8 (live); 19.3 ± 3.1 (post-mortem)	Commercial diet with vitamin–mineral premix	Post-mortem samples showed lower 25(OH)D_3_ concentrations	[89]

**Table 6:** Summary of studies evaluating vitamin D status in cattle.

Assay	25(OH)D Metabolites (ng/mL)	Diet/Vitamin D Source	Key Finding	Reference
HPLC	Mean ± SD: 36.9 ± 7.1	Vitamin D supplementation (NRC recommendations)	Reference 25(OH)D_3_ concentrations in outdoor cattle.	[85]
HPLC	~19 (winter); ~49 (pasture summer); ~21 (indoor summer)	Vitamin D_3_ supplementation (winter)	UVB exposure is the main determinant of 25(OH)D status.	[90]
RIA	Mean ± SD: 15.2–16.7 (winter), 26.3 (spring), 46.6–63.8 (summer/autumn)	Pasture + commercial diet with vitamin D	Marked seasonal variation; winter decline despite supplementation.	[91]
HPLC-MS/MS (isotope dilution)	Mean total ± SD: 39.3 ± 2.6	Pasture-raised cattle	Reference plasma 25(OH)D concentrations established.	[79]
HPLC with UV detection	Initially: 2.8 ± 0.2 After 28 days: natural group 28.6 ± 3.1, udder cover 23.2 ± 1.5, rug 8.9 ± 1.8, rug + udder cover 6.0 ± 0.5	For 6 months without vitamin D_3_ supplementation and without access to sunlight. During the experiment: corn- and clover-based TMR without added vitamin D_3_; access to pasture for 5 h daily	25(OH)D_3_ levels were highest in cows without coverings and lower in cows wearing blankets, indicating that vitamin D_3_ synthesis occurs across the entire skin surface—including beneath the coat—rather than primarily in the sebum on the coat ingested during grooming.	[92]

**Table 7:** Summary of studies evaluating vitamin D status in horses.

Assay	25(OH)D Metabolites (ng/mL)	Diet/Vitamin D Source	Key Finding	Reference
HPLC	Mean ± SD: Winter: 1.90 ± 0.23; Summer: 2.43 ± 0.09	Hay and oats (winter); fresh grass, hay, oats, and a mineral–vitamin supplement (summer)	Higher 25(OH)D concentrations in summer than in winter	[93]
EIA	Range: Foals: 9.3–22.0 (Thailand), 9.5–19.2 (USA); Adults: 18.4–30.5 (Thailand), 14.3–37.2 (USA)	Grass, hay and concentrates (no vitamin D supplementation)	Lower 25(OH)D_3_ concentrations in foals than in older horses.	[95]
ELISA	Median (IQR): 17.42 (9.82–30.85); Spring: 18.02; Autumn: 15.83	Alfalfa hay; no pasture access	Lower 25(OH)D concentrations in autumn than in spring.	[94]
ID-LC-MS/MS	25(OH)D_3_: undetectable in 78% of samples; 25(OH)D_2_: the predominant form of 25(OH)D, with higher concentrations in spring and summer than in autumn and winter.	Pasture (grass ad libitum) + hay when pasture access is limited	No significant differences in 25(OH)D_2_ and 25(OH)D_3_ levels were observed between blanketed and unblanketed horses; seasonality played a greater role than the use of blankets.	[96]

**Table 8:** Summary of studies evaluating vitamin D status in sheep.

Assay	25(OH)D Metabolites (ng/mL)	Diet/Vitamin D Source	Key Finding	Reference
HPLC	Mean ± SD: 12.9 ± 5.3	NRC-balanced diet with vitamin D_3_ supplementation; outdoor housing	Vitamin D_2_ and 25(OH)D_2_ remained the predominant circulating forms despite sunlight exposure and vitamin D_3_ supplementation.	[85]
HPLC–MS/MS	Mean ± SD: 39.16 ± 0.72; Single: 40.80 ± 1.36; Twin: 40.40 ± 0.96; Triplet: 33.20 ± 1.80	Outdoor pasture grazing under commercial farming conditions	Ewes carrying triplets had significantly lower 25(OH)D concentrations than ewes carrying singletons or twins.	[97]
HPLC–MS/MS	Mean ± SD: UBF: 15.2 ± 0.5; IBF: 15.8 ± 0.6; Lleyn: 17.7 ± 0.6	Outdoor grazing under Scottish hill conditions	Breed affected vitamin D status; Lleyn sheep had higher 25(OH)D_3_ concentrations than Scottish Blackface sheep.	[98]
HPLC-MS/MS (isotope dilution)	Mean ± SD: 11.0 ± 0.8	Pasture grazing; no vitamin D supplementation	Reported mean 25(OH)D concentration in this small reference population; comparisons with other species should be interpreted cautiously.	[79]

**Table 9:** Summary of studies evaluating vitamin D status in poultry.

Assay	25(OH)D Metabolites (ng/mL)	Diet/Vitamin D Source	Key Finding	Reference
HPLC	Mean: Turkeys: 18.2 ± 7.9; Chickens: 25.3 ± 3.9	Indoor housing; NRC diet supplemented with vitamin D_3_	Vitamin D_3_ and 25(OH)D_3_ were predominant circulating forms; levels reflect dietary supplementation.	[85]
RIA	Mean: 13.3 ± 4.3 (D_3_ diet) → 42.5 ± 18 (25(OH)D_3_ diet)	Broilers; vitamin D_3_ vs. 25(OH)D_3_ supplementation	25(OH)D_3_ showed ~3× higher bioavailability than vitamin D_3_.	[99]
HPLC	Mean: 25.11 → 23.77 (no supplement); 31.49 → 28.81 (+25(OH)D_3_)	Broilers; with/without 25(OH)D_3_ supplementation	Supplementation increased circulating 25(OH)D and improved vitamin D status.	[100]

**Table 10:** Summary of studies evaluating vitamin D status in fish.

Assay	25(OH)D Metabolites (ng/mL)	Diet/Vitamin D Source	Key Finding	Reference
UHPLC–MS/MS	Control: <1.0 (LOD); 69.8 μg/kg: 0.38 ± 0.2; 687 μg/kg: 8.5 ± 2.3; 6854.3 μg/kg: 95.5 ± 2.3 (plasma)	Basal diet: 131 μg/kg vitamin D_3_ (5240 IU/kg); supplemented with 25(OH)D_3_ at 69.8, 687, or 6854.3 µg/kg; indoor rearing.	Dietary 25(OH)D_3_ dose-dependently increased plasma 25(OH)D, improved growth and feed conversion at higher doses, and enhanced vitamin D status without adverse effects.	[102]
HPLC	0 μg/kg: ~0–1; ~70 μg/kg: ~8–10; ~6800–7000 μg/kg: ~90–100 (plasma)	Diets contained vitamin D_3_ or 25(OH)D_3_ (200, 400, 800 μg/kg); indoor rearing.	Indoor trout showed negligible endogenous vitamin D_3_ synthesis and limited conversion of dietary D_3_ to 25(OH)D. Direct 25(OH)D_3_ supplementation effectively increased vitamin D status.	[103]

**Table 11:** Summary of studies evaluating vitamin D status in reptiles.

Assay	25(OH)D Metabolites (ng/mL)	Diet/Vitamin D Source	Key Finding	Reference
LC-MS/MS	Mean: LED: 42.9–74.8; UVBN: 44.0–62.3; UVB: 43.5–54.3	UVB exposure; LED, UVBN or UVB	Higher 25(OH)D_3_ in LED than UVB and UVBN groups	[110]
ID-XLC-MS/MS	Mean ± SD: 15.2 ± 3.2 → 24.4 ± 8.0	Dietary vitamin D_3_ + UVB exposure	Higher 25(OH)D_3_ with UVB exposure	[111]
CLIA	Range: 17.3–357	Natural UV exposure; wild iguanas, diet less influential	Wide variation in 25(OH)D; possible species-specific variation and associations with pigmentation and reproduction.	[112]

**Table 12:** Summary of studies evaluating vitamin D status in wild boars.

Assay	25(OH)D Metabolites (ng/mL)	Diet/Vitamin D Source	Key Finding	Reference
ELISA	Mean: 20.69 ± 24.99; Winter: 11.2; Summer: 34.8	Natural forage or vitamin D_3_-enriched feed (2000 IU/kg).	Season and vitamin D_3_ supplementation increased 25(OH)D concentrations.	[44]
HPLC	Supplemented: 5.76 (2.48–9.03); Non-supplemented: 2.46 (1.66–3.26)	Natural forage ± vitamin D_3_-enriched feed (≈2000 IU/day).	Supplementation increased 25(OH)D_3_; higher levels were associated with milder tuberculosis.	[116]
LC–MS/MS	Mean: Adult ♀: 7.08 ± 0.86; Adult ♂: 4.32 ± 1.17; Juvenile ♀: 2.04 ± 1.65; Juvenile ♂: 2.56 ± 1.22	Natural forage; no supplementation.	Adults had higher 25(OH)D_3_ concentrations than juveniles.	[89]
HPLC-MS/MS (isotope dilution)	Mean: Liver: 2.5 ± 0.8 nmol/kg	Natural forage; no supplementation.	Lower hepatic 25(OH)D was reported in wild boars than in domestic pigs in the reference study; differences in diet and management should be considered when interpreting this comparison.	[79]

**Table 13:** Summary of studies evaluating vitamin D status in red deer.

Assay	25(OH)D Metabolites (ng/mL)	Diet/Vitamin D Source	Key Finding	Reference
HPLC-MS/MS (isotope dilution)	Mean: Plasma: 5.1 ± 0.4; Liver: 5.4 nmol/kg (hepatic tissue)	Free-ranging deer; no vitamin D supplementation.	Established reference plasma and liver total 25(OH)D concentrations for free-ranging red deer and reported differences in 25(OH)D concentrations among the species examined.	[79]
HPLC	Supplemented: 36.65 (26.80–46.48); Non-supplemented: 3.06 (0.46–5.66)	Vitamin D_3_-enriched feed (~8000 IU/day) ad libitum vs. no supplementation.	Vitamin D_3_ supplementation markedly increased serum 25(OH)D_3_ concentrations. Deer with localized tuberculosis had higher 25(OH)D_3_ concentrations than those with generalized disease, suggesting an association between better vitamin D status and milder *Mycobacterium bovis* infection.	[116]

**Table 14:** Summary of studies evaluating vitamin D status in wild rabbits.

Assay	25(OH)D Metabolites (ng/mL)	Diet/Vitamin D Source	Key Finding	Reference
EIA	Mean: 3.3 (range: 0.3–7.1)	Wild rabbits; natural diet, no vitamin D supplementation.	Serum 25(OH)D concentrations were low in the studied wild rabbit population. No significant effects of season, month, year, or sex were observed, suggesting persistently low vitamin D status in the studied population.	[43]

## Data Availability

The raw data supporting the conclusions of this article will be made available by the authors on request.

## Abbreviations

The following abbreviations are used in this manuscript:

25(OH)D	25-hydroxyvitamin D, calcidiol
25(OH)D_3_	25-hydroxycholecalciferol
25(OH)D_2_	25-hydroxyergocalciferol
1,25(OH)_2_D	1,25-dihydroxyvitamin D, calcitriol
UVB	Ultraviolet B
VDR	Vitamin D Receptor
VDBP	Vitamin D-Binding Protein
LC-MS/MS	Liquid Chromatography-Tandem Mass Spectrometry
HPLC	High-Performance Liquid Chromatography
RIA	Radioimmunoassay
EIA	Enzyme immunoassay
CLIA	chemiluminescence immunoassay
PTH	Parathyroid hormone
pQCT	Peripheral Quantitative Computed Tomography
HPLC–MS/MS	High-Performance Liquid Chromatography–Tandem Mass Spectrometry
UHPLC–MS/MS	Ultra-High-Performance Liquid Chromatography–Tandem Mass Spectrometry
ID-LC-MS/MS	Isotope-dilution HPLC-MS/MS
ID-XLC-MS/MS	Isotope dilution on-line solid-phase extraction liquid chromatography tandem mass spectrometry
